# A protocol for capturing RNA-sensing innate immune receptors in multiple conformations by single-particle cryo-EM

**DOI:** 10.1016/j.xpro.2023.102166

**Published:** 2023-03-14

**Authors:** Wenshuai Wang, Olga Fedorova, Anna Marie Pyle

**Affiliations:** 1 Department of Molecular, Cellular and Developmental Biology, Yale University, New Haven, CT 06511, USA; 2 Howard Hughes Medical Institute, Yale University, New Haven, CT 06520, USA

**Keywords:** Immunology, Molecular Biology, Protein Biochemistry, Protein Expression and Purification, Structural Biology, Cryo-EM

## Abstract

Capturing different conformations of receptor proteins that are complexed with ligands by single-particle cryo-EM facilitates our understanding toward the mechanisms of ligand recognition and receptor activation cascades. Here, we present a protocol for capturing RNA-sensing innate immune receptors, such as RIG-I, in multiple conformations by single-particle cryo-EM. We describe steps for protein-ligand sample preparation, data acquisition, and image processing covering focused three-dimensional classification. This protocol can be adapted to capture the dynamic behavior of other receptors that can be stabilized.

For complete details on the use and execution of this protocol, please refer to Wang and Pyle (2022).^1^

## Highlights

• Detailed steps for purification of RNA-receptor complex
• Step-by-step approach for grid preparation and screening, data acquisition, and processing
• Strategies for capturing innate immune receptor complexes in multiple conformations

## Abstract (editor highlights)

Publisher’s note: Undertaking any experimental protocol requires adherence to local institutional guidelines for laboratory safety and ethics.

## Graphical abstract

**Figure:**
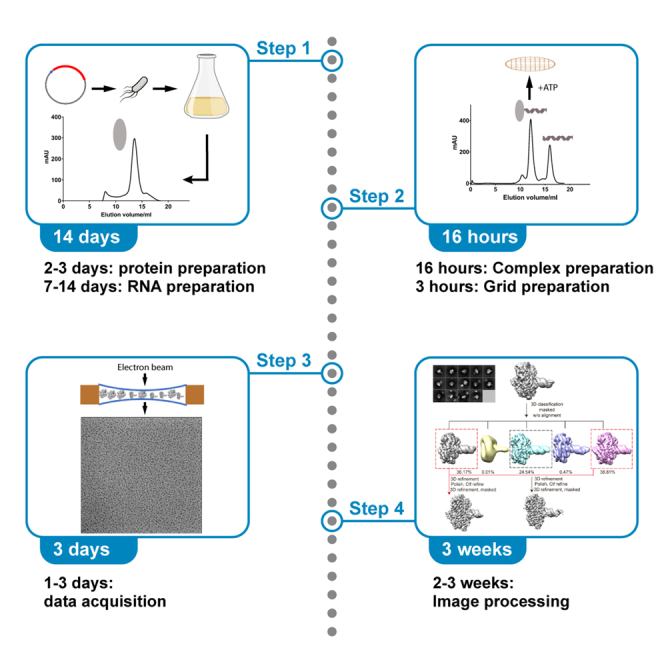

## Before you begin

The protocol below describes specific steps for capturing receptor conformations (in this case RIG-I (retinoic acid inducible gene-I)) in the presence of different stimulatory ligands (in this case viral and host double-stranded RNAs (dsRNAs)). These structures revealed that RIG-I utilizes two different conformations to distinguish viral and host dsRNA.^1^ This finding was achieved by collecting cryo-EM data from samples in the presence of viral or host dsRNAs, followed by data processing designed to isolate different conformations within the same dataset. The protocol below describes the sample preparation of making complexes of RIG-I and dsRNAs in the presence of ATP and Mg^2+^, followed by grid freezing. The single particle cryo-EM data acquisition and data processing are also described below.

### Protein preparation

**Timing: 2–3 days for protein preparation**

**Figure 1:**
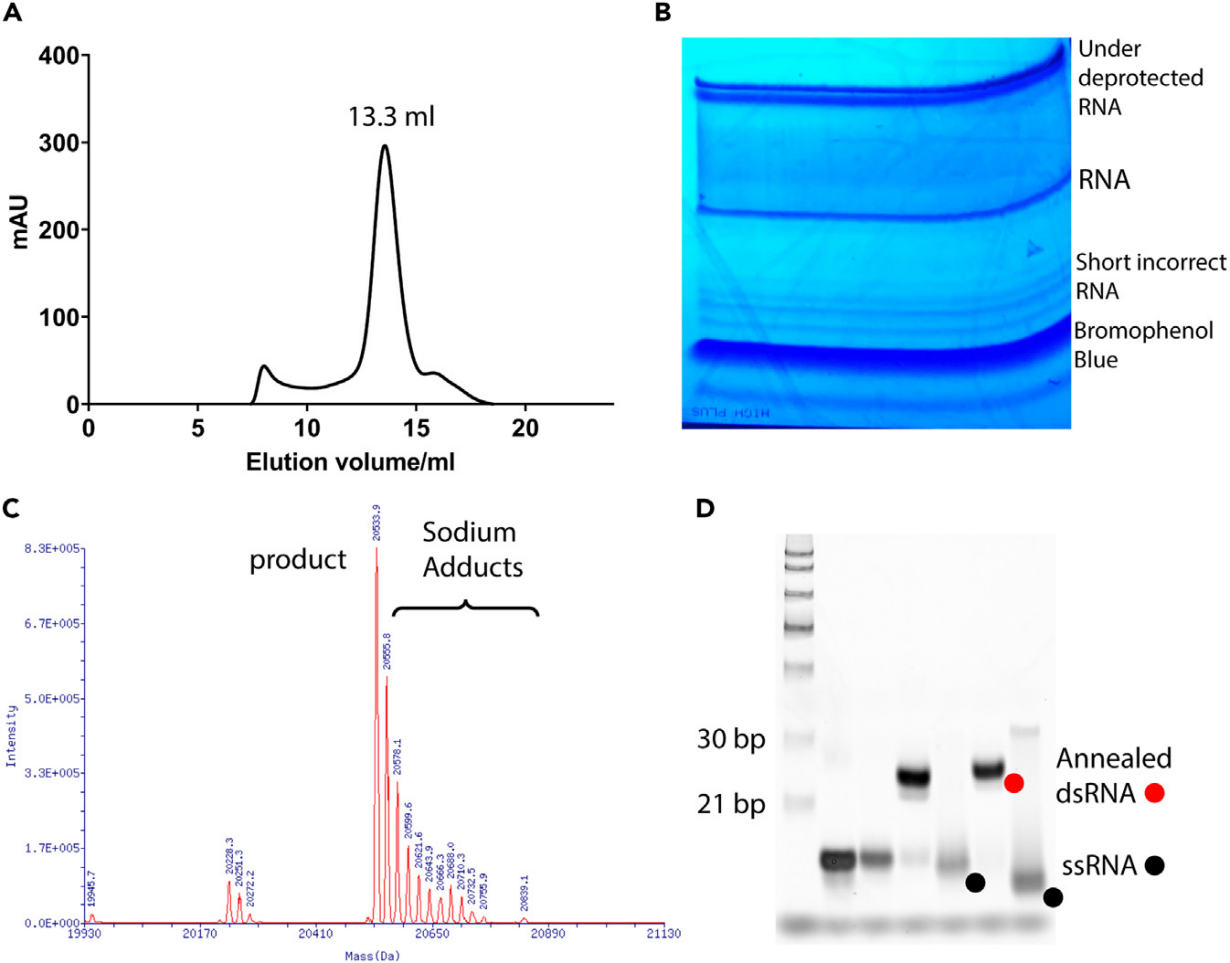
Representative results during protein and RNA preparation (A) Representative size-exclusion chromatography elution profile of human full-length RIG-I using Superdex 200 Increase 10/300 GL column. (B) Representative purification step of synthesized RNA oligonucleotide using 16% urea denaturing polyacrylamide gel. (C) Representative purity evaluation of ssRNA namely OHSLR30 using mass spec. The sodium adducts are OHSLR30 bound to multiple sodium ions. (D) Representative annealing evaluation of annealed dsRNA using 15% native gel.
  d. Pool RIG-I fractions in a storage buffer at concentration of 10–20 μM, flash freeze RIG-I in LN_2_ and store RIG-I at −80°C for further experiments.

1. Overexpress human RIG-I fused to an N-terminal 6×His tag and a SUMO tag, followed by ULP1 digestion site in ChampionTM pET SUMO vector in *E. coli* Rosetta™ 2(DE3) Singles™ Competent Cells. Troubleshooting 1.
  ***Note:*** N-Sumo tag improves the solubility and folding of RIG-I. In addition to the pET SUMO, other vectors containing the N-Sumo tag are also acceptable. The *E. coli* strain is Rosetta 2 (DE3) Singles Competent Cells (Millipore, 71400-3).
  a. Inoculate bacteria into 150 mL LB containing 30 μg/mL kanamycin and 25 μg/mL chloramphenicol.
    i. Incubate for 12–16 h at 37°C.
    ii. Add 25–35 mL cultured seeds to 1 L LB media containing antibiotics at same concentration as described above.
    iii. Add IPTG (Final, 0.5 mM; Stock, 1 M in ddH_2_O) to induce RIG-I overexpression when OD600 reaches 0.6, and proceed for 20–24 h at 16°C and 180 RPM.
  b. Collect cell pellets by centrifugation at 6,000 × *g* for 10 min, then put pellets to −80°C for storage.
    **Pause point:** The pellets can be stored at −80°C for 1 month, but fresh bacteria is recommended for protein purification.

2. Purify RIG-I by Nickel affinity chromatography.
  a. Weigh 10 g total bacteria pellets.
    i. Lyse them using a microfluidizer in the lysis buffer (10 mL/g) supplemented with EDTA-free Protease Inhibitor Cocktail.
    ii. Centrifuge cell lysate at 15,000 × *g* for 30 min and collect the supernatant.
  b. Equilibrate 3 mL of 50% slurry Nickle beads (by volume) with over 30 mL ddH_2_O and 30 mL lysis buffer, respectively.
  c. Mix the supernatant with the equilibrated beads, and stir the mixture at low speed, which does not produce abundant bubbles, at 4°C for 1 h.
  d. Pour the slurry to a gravity column. Wash beads with 20 mL wash buffer 1, followed by 20 mL wash buffer 2.
  e. Elute RIG-I with 10 mL elution buffer.
3. Purify RIG-I by heparin affinity chromatography and size-exclusion chromatography. Troubleshooting 1.
  a. Mix 20 mL lysis buffer with the 10 mL elution. Add 0.5 mg ULP1 to cleave SUMO tag from 2.5 mg eluted protein (yield from 10 g bacteria) at 4°C for 1 h or 12–16 h.
  b. Add 20 mL low salt buffer to 30 mL RIG-I solution.
    i. Then load the 50 mL solution onto a 5 mL HiTrap Heparin HP column at 2 mL/min at 4°C.
    ii. Elute RIG-I at 1 mL/min using 65% high salt buffer.
  c. Concentrate the eluted RIG-I to 0.5 mL using a 50 kD Amicon Ultra-4 centrifugal filter unit.
    i. Load RIG-I onto a Superdex 200 Increase 10/300 GL column with gel filtration buffer at 0.5 mL/min at 4°C.
    ii. Collect the peak fractions of monomeric RIG-I eluted around 13.3 mL (Figure 1A).

**Pause point:** The purified RIG-I can be stored at −80°C for at least one year.

### RNA preparation

**Timing: 7–14 days for RNA preparation**

4. To prepare RNA duplexes, synthesize RNA oligonucleotides (p3dsRNAa, p3dsRNAb; p2dsRNA; p1dsRNA; OHdsRNA) using an automated MerMade synthesizer (BioAutomation, Irving, TX, United States) with phosphoramidites from Glen Research using standard phosphoramidite chemistry.^2^
  a. Perform base deprotection to samples (1 mg of starting material) on the support in 2 mL 1:1 mixture (15% AMA, 20% MA) of 30% ammonium hydroxide (JT Baker) and 40% methylamine (Sigma) at 65°C for 10 min, vortex every 2 min.
    i. Cool the supernatant on ice for 10–15 min.
    ii. Transfer the supernatant to a new vial, freeze in −80°C for 45 min.
  b. Evaporate it in SpeedVac Vacuum Concentrators to dryness at 25°C for 3–4 h.
    i. Add 500 μL absolute ethanol.
    ii. Evaporate the solution to dryness 25°C for 30 min.
  c. In order to deprotect the 2′-OH groups, incubate the pellet with 500 μL of 1 M solution of tetrabutylammonium fluoride (TBAF) in Tetrahydrofuran (Sigma) at 25°C for 36 h.
  d. Add 500 μL 2 M sodium acetate (pH 6.0) and then evaporate the solution in SpeedVac Vacuum Concentrators at 25°C for 1 h to about 500 μL.
  e. Add 800 μL ethyl acetate, vortex and remove top layer of the solution, repeat three times, SpeedVac at 25°C for 15 min.
  f. Add 500 μL ddH2O to dissolve, add 2 mL absolute ethanol to precipitate at −20°C for 12–16 h.
  g. Purify RNA oligonucleotides using 16% urea denaturing polyacrylamide gel and assess the purity (>95% purity) by mass spectrometry (Novatia) (Figures 1B and 1C).
5. To prepare stem loop RNA (p3SLR30, OHSLR30), synthesize RNA oligonucleotides using in-vitro transcription.
  a. In-vitro transcribe RNA oligonucleotide using T7 RNA polymerase with synthetic dsDNA template (Integrated DNA Technologies) containing 2′-OMe modifications on the first two nucleotides of the 5′ terminus of the negative-sense strand.
  b. Prepare 100 μL transcription solution containing 1 μg of annealed template, 1 × transcription buffer, 10 mM DTT, 5 mM of each NTPs, 40 U of RNaseOUT™ Recombinant Ribonuclease Inhibitor (Thermo Fisher), and 5 μL of T7 RNA polymerase. Incubate the transcription solution at 37°C for 12 h.
  c. Purify transcribed RNA by gel extraction from 12%–20% urea denaturing polyacrylamide gel and assess its purity by mass spectrometry (Novatia) (Figures 1B and 1C).
  d. Treat p3SLR30 with calf intestinal alkaline phosphatase (CIP) to remove the triphosphate group. Assess OHSLR30 purity by mass spectrometry (Novatia) (Figure 1C).
6. Anneal RNA duplexes (p3dsRNA, p2dsRNA, p1dsRNA, OHdsRNA) and stem loop RNA (p3SLR30, OHSLR30).
  a. Anneal RNA duplexes (260 μM, 50 μL) by rapidly heating to 99°C and slowly cooling over 1 h to 4°C in the annealing buffer (200 mM NaCl) on a Thermocycler.
  b. Anneal stem loop RNA (60 μM) by heating to 90°C and incubate at 90°C for 2 min and then snap-cooled on ice for 30 min.
  c. Assess quality of annealed RNAs by running samples on a 15% native polyacrylamide gel (Figure 1D).

**Pause point:** The ssRNAs and dsRNAs can be stored in ddH_2_O at −80°C for at least one years.

## Key resources table

**Table:** 

REAGENT or RESOURCE	SOURCE	IDENTIFIER
**Chemicals, peptides, and recombinant proteins**		

Isopropyl β-D-thiogalactoside (IPTG)	AmericanBio	AB00841-00050
Chloramphenicol	Sigma-Aldrich	C0378-25G
Kanamycin	Sigma-Aldrich	10106801001
EDTA-free protease inhibitor cocktail	Sigma-Aldrich	11873580001
2-Mercaptoethanol	Sigma-Aldrich	M6250
SUMO protease (ULP1)	Self-made	N/A
T7 polymerase	Self-made	N/A
Adenosine 5′-triphosphate disodium salt hydrate	Sigma-Aldrich	A2383
Adenosine 5′-(β,γ-imido)triphosphate lithium salt hydrate	Sigma-Aldrich	A2647-25MG
Dithiothreitol (DTT)	Goldbio	DTT50
Spermidine	Sigma	85558-1G
Triton X-100	Sigma	93443-100ML
RNaseOUT™ Recombinant Ribonuclease Inhibitor	Thermo Fisher	10777019

**Bacterial and virus strains**		

Rosetta 2 (DE3) Singles Competent Cells	Millipore	71400-3

**Recombinant DNA**		

pET-SUMO-humanRIG-I	Ren et al. and Vela et al.^3^^,^^4^	N/A

**Critical commercial assays**		

Quick CIP	NEB	M0525S

**Oligonucleotides**		

ssDNA oligonucleotides	IDT	N/A
T7-SLR30-F	5′-TAATACGACTCACTATAGGATCGATCGA TCGATCGGCATCGATCGGCTTCGGCCGAT CGATGCCGATCGATCGATCGATCC-3′	N/A
T7-SLR30-R	5′-mGmGATCGATCGATCGATCGGCATCG ATCGGCCGAAGCCGATCGATGCCGATCG ATCGATCGATCCTATAGTGAGTCGTATTA-3′	N/A
ssRNA oligonucleotides	Self-made	N/A
p3dsRNAa	5′ppp-GGACGUACGUUUCGCGACUGUAGA-OH3′	N/A
p3dsRNAb	5′ppp-UCUACAGUCGUUCGACGUACGUCC-OH3′	N/A
p2dsRNA	5′pp-GGACGUACGUCGCGACGUACGUCC-OH3′	N/A
p1dsRNA	5′p-GGACGUACGUCGCGACGUACGUCC-OH3′	N/A
OHdsRNA	5′OH-GGACGUACGUCGCGACGUACGUCC-OH3′	N/A
p3SLR30	5′ppp-GGAUCGAUCGAUCGAUCGGCAUCGA UCGGCUUCGGCCGAUCGAUGCCGAUCGAU CGAUCGAUCC-OH3′	N/A
OHSLR30	5′OH-GGAUCGAUCGAUCGAUCGGCAUCGAUC GGCUUCGGCCGAUCGAUGCCGAUCGAUCGAU CGAUCC-OH3′	N/A

**Software and algorithms**		

GraphPad Prism 7	GraphPad	SCR_002798; http://www.graphpad.com/scientificsoftware/prism
SerialEM	Mastronarde^5^	SCR_017293; http://bio3d.colorado.edu/SerialEM/
MotionCor2	Zheng et al.^6^	SCR_016499; https://msg.ucsf.edu/em/software/motioncor2.html
CTFFinder4	Rohou and Grigorieff^7^	SCR_016732; https://grigoriefflab.umassmed.edu/ctffind4
Relion 3.0	Zivanov et al.^8^	SCR_016274; https://www3.mrc-lmb.cam.ac.uk/relion/index.php/Download_%26_install
Relion 4.0	Kimanius et al.^9^	https://relion.readthedocs.io/en/release-4.0/Installation.html
UCSF Chimera	Pettersen et al.^10^	SCR_004097; https://www.cgl.ucsf.edu/chimera/
Phenix 1.17.1_3660	Afonine et al.^11^	SCR_014224; https://www.phenix-online.org/
Coot 0.8.9	Emsley et al.^12^	SCR_014222; https://www2.mrc-lmb.cam.ac.uk/personal/pemsley/coot/
Pymol 2.5.0a0	Schrödinger, LLC	SCR_000305; https://pymol.org/2/
MolProbity	Williams et al.^13^	SCR_014226; http://molprobity.biochem.duke.edu/

**Deposited data**		

p3dsRNA:RIG-I complex	EMDB, PDB	EMDB: EMD-26022, PDB: 7TNX
p2dsRNA:RIG-I complex	EMDB, PDB	EMDB: EMD-26023, PDB: 7TNY
p1dsRNA:RIG-I complex	EMDB, PDB	EMDB: EMD-26024, PDB: 7TNZ
OHdsRNA:RIG-I complex	EMDB, PDB	EMDB: EMD-26025, PDB: 7TO0
p3SLR30 end bound complex	EMDB, PDB	EMDB: EMD-26026, PDB: 7TO1
p3SLR30 internally bound complex	EMDB, PDB	EMDB: EMD-26027, PDB: 7TO2
OHSLR30 end bound complex	EMDB, PDB	EMDB: EMD-27744, PDB: 8DVS
OHSLR30 internally bound complex	EMDB, PDB	EMDB: EMD-27745, PDB: 8DVU
p3dsRNA:RIG-I complex (+AMPPNP)	EMDB, PDB	EMDB: EMD-27743, PDB: 8DVR

**Other**		

Quantifoil holey carbon R1.2/1.3 300 mesh Cu grids	Ted Pella	https://www.tedpella.com/calibration_html/QUANTIFOIL_TEM_Substrates.htm
Ni-NTA Superflow beads	Qiagen	30230
Econo-Pac® Chromatography Columns	Bio-Rad	7321011
HiTrap Heparin HP column	GE Healthcare	GE17-0407-01
Superdex 200 Increase 10/300 GL column	GE Healthcare	28990944
SpeedVac Vacuum Concentrators	Thermo Fisher	N/A
PELCO easiGlow™ Glow Discharge Cleaning System	Ted Pella	N/A
FEI Vitrobot™ Mark IV	ThermoFisher	N/A
Glacios transmission electron microscope operating at 200 keV and equipped with a Gatan K2 Summit direct electron detector.	Thermo Fisher	N/A
Titan Krios transmission electron microscope operating at 300 keV and equipped with a Gatan K3 Summit direct electron detector.	Thermo Fisher	N/A

## Materials and equipment

**Table:** Lysis buffer

Reagent	Final concentration	Amount
HEPES (0.5 M), pH 8.0	25 mM	50 mL
NaCl (5 M)	300 mM	60 mL
Glycerol (100%)	10% v/v	100 mL
BME (14.3 M)	5 mM	350 μL
ddH_2_O	N/A	790 mL
**Total**	**N/A**	**1,000 mL**

***Note:*** The pH is adjusted with 3 M NaOH. The buffer without BME is stored at 4°C for up to 3 months. BME is freshly added before usage.

**Table:** Wash buffer 1

Reagent	Final concentration	Amount
HEPES (0.5 M), pH 8.0	25 mM	1 mL
NaCl (5 M)	1 M	4 mL
Glycerol (100%)	10% v/v	2 mL
BME (14.3 M)	5 mM	7 μL
Imidazole (4 M), pH 8.0	30 mM	150 μL
ddH_2_O	N/A	11.8 mL
**Total**	**N/A**	**20 mL**

***Note:*** The pH is adjusted with 3 M NaOH. The buffer without BME and Imidazole is stored at 4°C for up to 3 months. BME and Imidazole are freshly added before usage.

**Table:** Wash buffer 2

Reagent	Final concentration	Amount
Lysis buffer	1 ×	19.9 mL
Imidazole (4 M), pH 8.0	20 mM	100 μL
BME (14.3 M)	5 mM	7 μL
**Total**	**N/A**	**20 mL**

***Note:*** The buffer is freshly made with lysis buffer. BME and Imidazole are freshly added before usage.

**Table:** Elution buffer

Reagent	Final concentration	Amount
Lysis buffer	1 ×	9.375 mL
Imidazole (4 M), pH 8.0	250 mM	625 μL
BME (14.3 M)	5 mM	3.5 μL
**Total**	**N/A**	**10 mL**

***Note:*** The buffer is freshly made with lysis buffer. BME and Imidazole are freshly added before usage.

**Table:** Low salt buffer

Reagent	Final concentration	Amount
HEPES (0.5 M), pH 7.4	25 mM	50 mL
Glycerol (100%)	10% v/v	100 mL
BME (14.3 M)	5 mM	350 μL
ddH_2_O	N/A	850 mL
**Total**	**N/A**	**1,000 mL**

***Note:*** The pH is adjusted with 3 M NaOH. The buffer without BME is stored at 4°C for up to 3 months. BME is freshly added before usage.

**Table:** High salt buffer

Reagent	Final concentration	Amount
HEPES (0.5 M), pH 7.4	25 mM	25 mL
NaCl (5 M)	1 M	100 mL
Glycerol (100%)	10% v/v	50 mL
BME (14.3 M)	5 mM	175 μL
ddH_2_O	N/A	325 mL
**Total**	**N/A**	**500 mL**

***Note:*** The pH is adjusted with 3 M NaOH. The buffer without BME is stored at 4°C for up to 3 months. BME is freshly added before usage.

**Table:** Gel filtration buffer and storage buffer

Reagent	Final concentration	Amount
HEPES (0.5 M), pH 7.4	25 mM	25 mL
NaCl (5 M)	200 mM	20 mL
Glycerol (100%)	5% v/v	25 mL
BME (14.3 M)	5 mM	175 μL
ddH_2_O	N/A	430 mL
**Total**	**N/A**	**500 mL**

***Note:*** The pH is adjusted with 3 M NaOH. The buffer without BME is stored at 4°C for up to 3 months. BME is freshly added before usage.

**Table:** 10 × transcription buffer

Reagent	Final concentration	Amount
Tris-HCl (1 M), pH 8.0	400 mM	400 μL
MgCl_2_ (2 M)	220 mM	110 μL
Spermidine (2 M)	20 mM	10 μL
Triton X-100	0.1%	0.01%
ddH_2_O	N/A	480 μL
**Total**	**N/A**	**1 mL**

***Note:*** The pH is adjusted with 12 M HCl. The buffer is stored at 4°C for up to 1 month.

**Table:** *In vitro* transcription reaction solution

Reagent	Final concentration	Amount
10 × transcription buffer	1 ×	10 μL
DTT (1 M)	10 mM	1 μL
Each NTPs (30 mM), pH 7.0	5 mM	16.7 μL × 4
RNaseOUT™ Recombinant Ribonuclease Inhibitor (40 U/ μL)	40 U	1 μL
T7 RNA polymerase (2 mg/mL)	0.1 mg/mL	5 μL
ddH_2_O	N/A	16.2 μL
**Total**	**N/A**	**100 μL**

***Note:*** The pH of individual NTPs solution is adjusted to 7.0 with 3 M NaOH. Incubate the transcription solution at 37°C for 12 h.

## Step-by-step method details

### Sample preparation for freezing grids

**Timing: 16 h**

These steps describe the purification of RNA complexes and preparation for freezing grids.

1. Mix RIG-I with 24-basepaired p3dsRNA, p2dsRNA, p1dsRNA, OHdsRNA, 30-basepaired p3SLR30 and OHSLR30 in an 8:1, 2:1, 1.5:1, 2:1, 1:1 and 1:1 M ratio, respectively, followed by incubation at 4°C for 12 h.

***Note:*** It is well established that the footprint size of RIG-I on dsRNA is 8–12 bp. We found that lengthening the RNA stem (14 bp to 24 bp) had several technical advantages and it improved our ability to image the particles due to the inherently high contrast of RNA duplexes (Global resolution was improved from 3.8 Å to 3.5 Å).

2. Purify the RIG-I:RNA complex by loading the 500 μL solution to Superdex 200 Increase 10/300 GL column with gel filtration buffer (without glycerol).
3. Concentrate the p3dsRNA:RIG-I, p2dsRNA:RIG-I, p1dsRNA:RIG-I, OHdsRNA:RIG-I, p3SLR30:RIG-I and OHSLR30:RIG-I complexes to 0.3, 1.5, 0.4, 1.5, 1.5 and 1.5 mg/mL using 50 kD Amicon Ultra-0.5 centrifugal filter unit.

**Figure 2:**
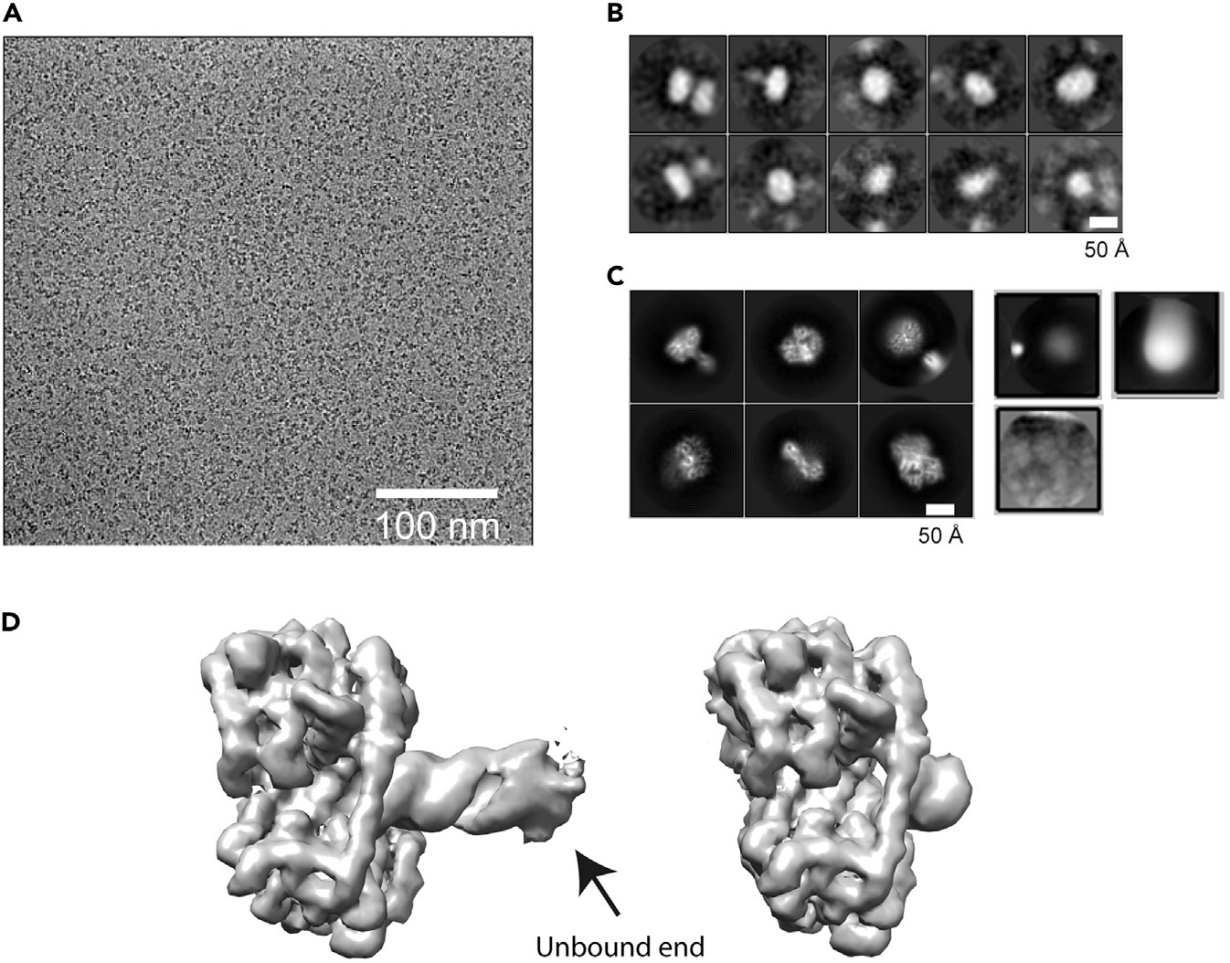
Representative data during data collection and processing (A) Representative micrograph. (B) Representative 2D class averages using manual pick particles. (C) Representative 2D class averages showing good and bad features. The 1:1 and 2:1 RIG-I:RNA complexes are rendered. The representative 2D class averages of bad particles (upper left), ice contamination (upper right) and carbon (lower left) are shown. (D) Representative 3D maps of intact 1:1 RIG-I:RNA complex and unbound end-removed complex.

***Note:*** These concentrations are used because they achieve decent particle density on grid (Figure 2A).

4. In the case of p3SLR30:RIG-I complex with ATP in solution, OHSLR30:RIG-I complex with ATP in solution and p3SLR30:RIG-I with AMPPNP in solution, concentrate the p3SLR30:RIG-I and OHSLR30:RIG-I complexes to 3.0 and 2.5 mg/mL, respectively. Incubate 20 μL of 3, 20, 20 μM (0.3, 2.3, 2.3 mg/mL) of complex with 2.5 mM ATP/AMPPNP and 5 mM MgCl_2_ on ice for 30 min before applying samples to the grids.

**CRITICAL:** Fresh samples are recommended for freezing grids, as this may help improve the resolution of cryo-EM maps.

***Note:*** These concentrations are used because they achieve good particle density on grids (Figure 2A).

### Cryo-EM grid preparation

**Timing: 3 h**

These steps describe the preparation of cryo-EM grids for gird screening and data collection.

***Note:*** The course is available at link: https://emcore.ucsf.edu/content/vitrobot-markiv-notes. The steps below might be different from the course.

5. With the purified samples on ice, mount the humidifier to Vitrobot Mark IV with O-ring well seated on top of the humidifier.
  a. Add 50 mL distilled ddH_2_O to the humidifier.
  b. Install two blot papers (Whatman no. 1) on each side.
  c. Turn on the Vitrobot.
6. Set up the temperature to 22°C and the humidity to 100%.
  a. Assemble the dry cryogen container (liquid nitrogen reservoir, float, brass cryogen cup, metal storage box holder, metal “spider legs” and labeled grid boxes).
  b. Cool the container by filling with liquid nitrogen (LN_2_) to the brass cryogen cup.
  c. After the container is cooled, fill the reservoir with LN_2_ again, but do not add to the brass cup.

***Note:*** After the container is cooled, make sure the brass cryogen cup does not contain LN_2_.

7. Glow discharge the Quantifoil holey carbon R1.2/1.3 300 mesh Cu grids with carbon side facing up using the PELCO easiGlow™ Glow Discharge Cleaning System (Ted Pella) for 35 s at 25 mA. Troubleshooting 2.
8. Fill the brass cryogen cup with ethane when the grids are ready for usage.

**CRITICAL:** Ethane can evaporate and form flammable gas. Close the valve of ethane after usage and pay attention to the ethane monitor. Dispose the used ethane in fume hood. Do not work with ethane near heat and fire.

***Note:*** As liquid ethane in cryogen cup will solidify in a short time, it is recommended to fill with liquid ethane right before freezing grids.

9. Hold one grid with Vitrobot tweezer and mount the tweezer to Vitrobot.
  a. Lift the tweezer with grid, then transfer and lift the container.
  b. Apply 3 μL samples to the carbon side of the grid, blot samples with force -4 for seconds (2, 3, 4, 5 s), followed by plunging grid to liquid ethane.
  c. Transfer the grid to one of the four slots of the grid box. Troubleshooting 3.
10. Repeat step 9 with adjusted blotting time to freeze more grids. Troubleshooting 4.
11. Clip the frozen grids using the C-clip and the C-clip ring in the grid clipping station (ThermoFisher), and store the autogrids (clipped grids) in the autogrid boxes.

***Note:*** Sometimes solid ethane covers the grid surface. To prevent the solid ethane from damaging the grid during grid clipping, the grids can be left in LN_2_ for 1–2 days to remove the solid ethane.

12. Transfer the autogrid boxes to the LN_2_ storage dewar for grid screening and data collection.

**Pause point:** The grids can be stored in LN_2_ dewar for months before loading to electron microscope for grid screening and data collection, but it is better to restrict it within 2 months.

### Cryo-EM data acquisition

**Timing: 1–3 days**

These steps describe the grid screening and cryo-EM data collection.

13. Mount the grids to the Glacios using the autoloader and screen the grids.
  a. Take a full map of the grids using the software SerialEM^5^ and check the ice thickness.
  b. Select grids with thin ice and pick 2–3 squares with ice of different thickness.
    ***Note:*** The squares where most holes are visible but the square edges are not sharp usually have decent ice thickness for grid screening and data collection.
    i. Select 2–3 holes in one square, take micrographs from either center or edge of the holes.
    ii. Check particle quality, particle density, ice thickness and crystalline ice contamination.
14. Collect micrographs from the qualified grids (Table 1, Figure 2A). Troubleshooting 5.
  ***Alternatives:*** Both Glacios and Krios are suitable to collect high-quality data for reconstructing high-resolution cryo-EM maps.
  a. Set up the data collection conditions, make sure the beam is ready for high-resolution data collection.
  b. Collect the micrographs with calibrated pixel size of around 1 Å/pix and total electron exposure of 50–60 e^-^/Å^2^ (total electron exposure/frame, 1–1.5 e^-^/Å^2^).
  c. Use different defocus ranges during data acquisition (Table 1).
    ***Alternatives:*** When the particles are still visible by eyes under low defocus range (low, -0.8∼-2.0 μm; high -1.2∼-3.0 μm), low defocus range can be applied to improve the resolution.
  d. For each stage movement, acquire one micrograph/hole of 9 holes on R1.2/1.3 grid.
    ***Alternatives:*** For R2/2 grid, three to five micrographs/hole can be collected.
  e. Collect about 3000 micrographs under super-resolution mode in one day. Generate the jpg picture during data collection for monitoring the image quality.
    ***Alternatives:*** The required number of micrographs depends on number of autopicked particles required for data processing (1 million particles are a good start.).

**Table 1:** Cryo-EM data collection and image processing statistics

Structure:	OHdsRNA:RIG-I	p1dsRNA:RIG-I	p2dsRNA:RIG-I	p3dsRNA:RIG-I	p3SLR30:RIG-I+ATP		OHSLR30:RIG-I+ATP			p3SLR30: RIG-I+ AMPPNP
					Internally-bound	End-bound	Internally-bound 1	Internally-bound 2	End-bound	p3SLR30:RIG-I
**EMDB**	EMD-26025	EMD-26024	EMD-26023	EMD-26022	EMD-26027	EMD-26026		EMD-27745	EMD-27744	EMD-27743

**Data collection and processing:**										

Voltage (KeV)	300	300	300	300	300		200			300
Pixel size (Å)	1.068	1.068	1.068	1.05	1.05		1.149			1.068
Magnification	81,000 ×	81,000 ×	81,000 ×	130,000 ×	130,000 ×		36,000 ×			81,000 ×
Defocus range (μm)	-1.2 ∼ -3.0	-1.2 ∼ -3.0	-1.2 ∼ -3.0	-1.2 ∼ -2.7	-1.2 ∼ -2.7		-0.8 ∼ -2.0			-1.2 ∼ -3.0
Total electron exposure (e^-^/Å^2^)	59	59	59	61	55		50			60
Dose rate (e^-^/pix/s)	7.9	17.7	17.7	17.7	9.4		6.3			18
Exposure time (s)	8.5	3.8	3.8	3.8	8.0		10.5			3.8
Frames per micrograph	34	38	38	38	40		53			38
Collected micrographs	2838	2460	2586	3480	3417		3663			2744
Initial particle images (no.)	3,357,160	3,005,433	3,354,308	1,996,263	2,188,358		3,635,886			3,097,952
Final particle images (no.)	435,184	624,117	957,706	613,519	366,751	253,073	1,224,061	631,595	386,764	692,833
Symmetry applied	C1	C1	C1	C1	C1	C1	C1	C1	C1	C1
Map resolution (Å)	3.5	3.5	3.2	3.5	3.2	3.7	3.0	2.9	3.0	3.3
FSC threshold	0.143	0.143	0.143	0.143	0.143	0.143	0.143	0.143	0.143	0.143
Local resolution range (Å)	2.3–5.0	2.3–5.0	2.3–5.0	2.3–5.0	2.3–5.0	2.3–5.0	2.5–5.0	2.5–5.0	2.5–5.0	2.3–5.0
Sharpening B-factor (Å^2^)	-146.58	-189.05	-126.40	-150.00	-82.00	-140.00	-116.23	-114.44	-118.35	-157.95

### Cryo-EM data processing

**Timing: 2–3 weeks**

These steps describe the process of reconstructing high-resolution maps of different conformations (Table 1).

15. Process datasets through Relion.^8^ The micrographs are dose-weighted and beam induced motion corrected through MotionCor2.^6^
  a. With 5 × 5 patches, bin twice with micrographs collected under super-resolution mode.
  b. Select micrographs with total motion below 40 Å for CTF estimation.
16. Estimate the CTF parameters using CTFFIND4^7^ with the non-dose-weighted and motion corrected micrographs.
  a. Estimate the CTF of selected micrographs with FFT box size of 1024 pix.
  b. Select the micrographs with resolution above 6 Å.
  c. Manually evaluate the selected micrographs; Dispose of micrographs with bad CTF fitness of Thon rings and ice contamination.

**Figure 3:**
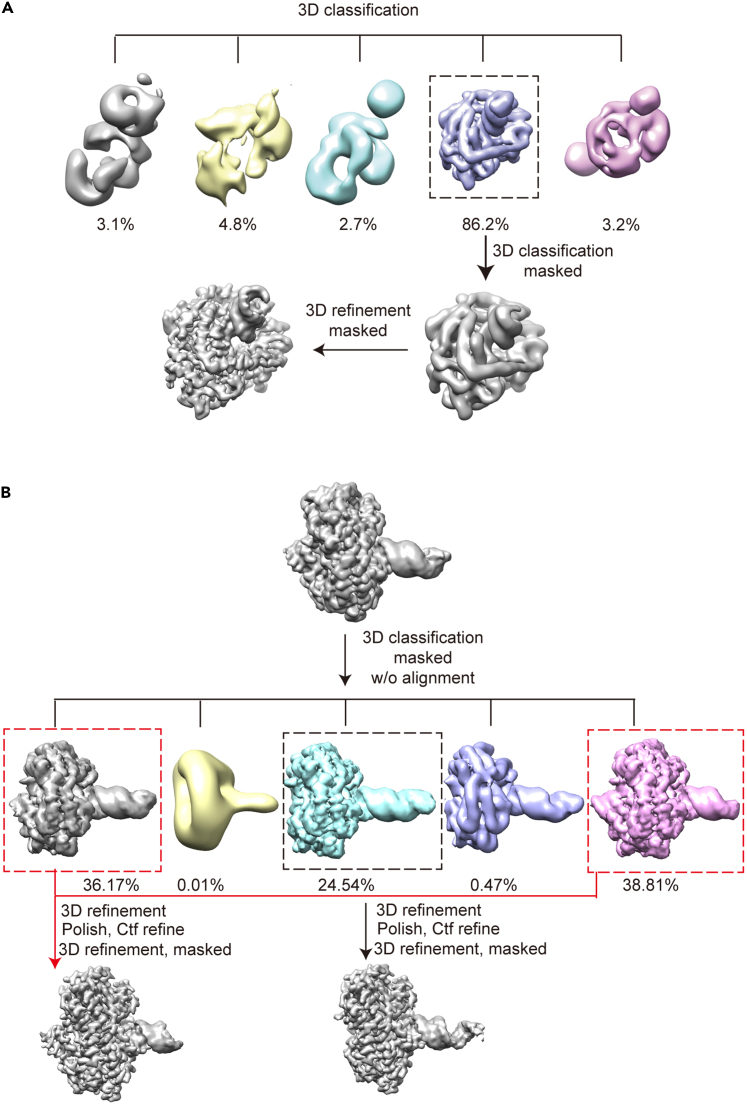
Workflow of improving global resolution and identifying different conformational states using focused 3D classification w/i and w/o alignment (A) Focused 3D classification w/i alignment using mask to exclude the unbound RNA end. (B) Focused 3D classification w/o alignment using mask covering the whole EM map.
17. Pick up particles from selected micrographs using autopick.
  a. Generate references for autopick.
    i. Manually pick up 2,000–3,000 particles from 100 micrographs.
      ***Note:*** Adjust the scale and sigma of micrographs to help pick up particles. Sometimes small scale and sigma help people easily figure out the single particles.
    ii. Perform 2D classification with the manually picked particles using 10–20 classes.
    iii. Select the 2D classes showing features of complex (Figure 2B).
      ***Alternatives:*** The previously resolved cryo-EM map of the sample can be used as the 3D reference of autopick. The pixel size and box size of the map should be adjusted to the same as the binned micrographs using relion_image_handler command.
  b. Autopick particles from micrographs.
    i. Optimize the “Picking threshold”, “Minimum inter-particle distance” and “Maximum stddev noise” (1, 100, 1.1) to pick up nearly all the particles.
    ii. Manually assess the autopick results. Make sure most of the particles are picked up; Unpick the ice and carbon.
      ***Alternatives:*** The Gautomatch (developed by Prof. Kai Zhang, https://www2.mrc-lmb.cam.ac.uk/download/gautomatch-056/) can also be used for autopicking if Relion autopick fails to pick up most of the particles possibly due to low defocus for data collection.

18. Perform 2D classification of the autopicked particles.
  a. Downscale the particles by a factor of two using Extract job to accelerate data processing and save computational resources.
  b. Perform 2D classification using 100 classes.
  c. Select classes with features of particles and dispose of classes representing misfolded samples or ice contamination (Figure 2C).
    ***Note:*** Only discard classes that are certainly contamination. The bad particles derived from seemingly bad 2D averages can be removed later during 3D classification in step 19.
  d. Carry on 2–3 rounds of 2D classification.
  e. Select particles from all 2D classes containing a 1:1 RNA:RIG-I complex and from 2D classes showing 1:2 RNA:RIG-I complex in top and bottom views (Figure 2C).
    ***Note:*** We notice there are more side views of 1:1 particles. To alleviate the preferred orientation, we purposely select classes of 1:2 particles with top and bottom views.

19. Generate the initial model with selected binned particles and perform several rounds (2–3) of 3D classification through Relion (Figure 3A). Troubleshooting 6.
  a. Generate a single initial map using Relion 3D initial model (Mask diameter, 200 Å; Flatten and enforce non-negative solvent, Yes; Symmetry, C1; Number of initial iterations, 50; Number of in-between iterations, 200; Number of final iterations, 50), and use it as the 3D reference of 3D classification (3–5 classes; Initial low-pass filter, 60 Å; Regularization parameter T, 4; Number of iterations, 25; Mask diameter, 200 Å). Select the best 3D class.
  b. To improve the resolution, erase the unbound end of dsRNA in the 3D map generated above through Chimera,^10^ and apply a soft mask (Lowpass filter map, 15 Å; Initial binarization threshold, 0.002; Extend binary map this many pixels, 6; Add a soft-edge of this many pixels, 6) to the modified map to re-implement the 3D classification with selected particles from the 2D classes (Figure 2D).
  c. With particles from the best 3D class, perform masked 3D refinement (Initial low-pass filter, 60 Å; Use solvent-flattened FSCs, Yes), Ctfrefine (Minimum resolution for fits, 30 Å; Perform CTF parameter fitting, Yes; Fit per-particle defocus, Yes; Range for defocus fit, 2,000 Å; Perform beamtilt estimation, Yes) and Byaesian polishing (Optimized parameters using 10,000 particles, then use the optimized parameters to polish micrographs), followed by a final 3D refinement.^8^^,^^14^
    ***Note:*** For samples containing multiple conformations, particles from the best 3D class could be applied to another 3D classification (5–10 classes, T = 4) without alignment, thereby classifying particles into 3D classes of different conformations. Then these maps of different conformations can be subjected to subsequent 3D refinement etc (Figure 3B).
  d. Perform postprocessing job to yield maps at near-atomic global resolution ranging from 2.9 Å to 3.7 Å (Table 1) according to the FSC = 0.143 criterion and sharpened with B-factor.^15^

## Expected outcomes

In this step-by-step protocol, we described the detailed steps of sample preparation, grid freezing, data acquisition and data processing. Following this protocol, we have successfully reconstructed the cryo-EM maps of RIG-I in different conformations in the presence of different dsRNAs and ATP.^1^ This protocol can be further utilized to explore structures of RIG-I:RNA complexes from heterogeneous samples, and adapted to study other similar receptors.

## Quantification and statistical analysis

All the datasets were processed using Relion, assisted with Chimera.

## Limitations

Although this protocol was successfully used to explore different RIG-I conformations, it may not be utilized to study other macromolecular complexes without adaptation, since each complex shows distinctive characteristics. To capture the sub-state conformations, it’s more worthwhile stabilizing the complex in these conformations by biochemistry methods, such as assembly with other or more binding partners, introduction of truncations and mutations, addition of compounds and change of buffer conditions. When these sub-state conformations stably exist in solution, it is expected to be feasible to use the cryo-EM technique in this protocol to resolve these structures, otherwise, the time-resolved cryo-EM technique might be a better option capturing the transient unstable sub-state conformations.

## Troubleshooting

### Problem 1

Fail to obtain monomeric RIG-I protein. Refer to steps 1 and 3 of protein preparation section.

### Potential solution

Inducing RIG-I expression at high OD600 might lead to more RIG-I aggregates. It is good practice to reduce RIG-I aggregates by inducing RIG-I expression when OD600 achieves 0.6.

The His tagged SUMO tag might be removed before recovering samples with Ni Beads. This can be resolved by using fresh bacteria or short-term (one month) stored bacteria at −80°C.

### Problem 2

There are few particles per micrograph. Refer to step 7 of cryo-EM grid preparation.

### Potential solution

To trap more particles in the hole, it is worth trying to increase sample concentration, testing different grids, such as grids of different brands (quantifoil, c-flat), grids with different support (Cu, Au), grids without carbon (UltraAu), or grids coated with a single carbon or graphene oxide layer. Reverse blotting (glow discharging on one side and applying samples to the other side) might help as well.

### Problem 3

Ice contamination including crystalline ice. Refer to step 9 of cryo-EM grid preparation.

### Potential solution

Make sure the cryogen container is cooled thoroughly. Be careful when transferring frozen grids from liquid ethane to LN_2_ and from Autogrid box to autoloader. The transient exposure to ambient temperature might cause thaw-freeze cycle which usually leads to crystalline ice. Use clean fresh LN_2_ can reduce chunk of ice as well.

### Problem 4

Ice is either too thin or too thick. Refer to step 10 of cryo-EM grid preparation.

### Potential solution

It is hard to reproduce the grid even under same freezing conditions. To freeze grids with optimal ice thickness, it is recommended to freeze a series of grids using different blotting time on the same batch.

### Problem 5

Poor data quality of micrographs. Refer to step 14 of cryo-EM data acquisition.

### Potential solution

During data acquisition, keep an eye on the collected micrographs. If the micrographs show abnormal features, such as small file size and incomplete view, stop data collection and check if the microscope works as expected.

### Problem 6

Only poor 3D class maps are obtained after 3D classification. Refer to step 19 of cryo-EM data processing.

### Potential solution

To obtain a good 3D map from a dataset for the first time, sometimes this requires multiple rounds of 3D classification (10 rounds). After the 3D map with features of secondary structures shows up, this map can be used as 3D reference of 3D classification with selected particles from 2D classification. This time it only requires one or two rounds of 3D classification.

If there is a reliable high-quality cryo-EM map of samples similar to the complex, this map can be used as 3D reference of 3D classification.

If all these above do not work, review the autopick and 2D classification jobs, and check whether most of the particles are picked up and whether 2D classes show clear features of secondary structures.

If the particles are picked up properly but 2D classes don’t have distinctive features, the sample should be further optimized.

## Resource availability

### Lead contact

All requests for resources and reagents should be directed to and will be fulfilled by the lead contact, Anna Marie Pyle (anna.pyle@yale.edu).

### Materials availability

All materials will be available from the lead contact with a completed Materials Transfer Agreement.

### Data and code availability

Atomic coordinates and cryo-EM maps are deposited in EMDB and PDB as described in key resources table and the published paper.^1^

This paper does not report original code.

Any additional information required to reanalyze the data reported in this paper is available from the lead contact upon request.
